# The Mystery and Romance of Alchemy and Pharmacy

**Published:** 1900-03

**Authors:** 


**The Mystery and Romance of Alchemy and Pharmacy.** By C. J. S. Thompson. Pp. xv., 335. London: The Scientific Press, Limited. 1897.

In many, if not most, medical libraries there is a small space devoted to the lighter side of literature, wherein may often be found volumes of anecdote relating to the history of the profession of medicine and its followers. In this not too conspicuous corner *The Mystery and Romance of Alchemy and Pharmacy* may well find a place.

The earliest authentic prescription, the mystic teachings of the Greek philosophers, the black art, the quack, and that symbolic instrument the mortar, all furnish texts for interesting discourses in the first section of the book. The second is occupied by well-remembered extracts from the writings of authors, from Chaucer to Marryat, who have portrayed the alchemist, the empiric, and their successors. Mr. Thompson has produced just the kind of book that is useful to "ease off" the strain of a long day's application to work before retiring to rest.

